# Laminin mediates tethering and spreading of colon cancer cells in physiological shear flow

**DOI:** 10.1038/sj.bjc.6690622

**Published:** 1999-08

**Authors:** J Kitayama, H Nagawa, N Tsuno, T Osada, K Hatano, E Sunami, H Saito, T Muto

**Affiliations:** Department of Surgery, Division of Surgical Oncology; Department of Transfusion Medicine; The Surgical Center, The University of Tokyo, Hongo 7-3-1, Bunkyo-ku, Tokyo 113, Japan

**Keywords:** metastasis, laminin, shear flow, α6 integrin

## Abstract

Under the physiological shear condition, cultured colon cancer cells bound to laminin (LM), but not to fibronectin or vitronectin. Most of the tethered cells did not roll, but arrested immediately and spread within 10–30 min on LM under the continuous presence of shear flow. The tethering of Colo201 was partially inhibited by monoclonal antibodies (mAbs) to α6 integrin and a combination of mAbs to β1 and β4 integrins, but not by mAb to 67KD laminin receptor. Some Colo201 cells still tethered at 4°C. This suggests that α6β1 and α6β4 integrins participate in Colo201 tethering on LM, although other non-integrin molecules play roles. In contrast, the spread of Colo201 was effectively inhibited by the mAbs to integrin α2, α6 and β1 chains. The effect of anti-α2 plus anti-α6 mAbs was almost equal to anti-β1, suggesting that Colo201 cells mainly use α2β1 and α6β1 integrins for spreading on LM. When the cells were perfused on subconfluent endothelial cells (HUVEC) cultured on LM, they did not tether on HUVEC but did on coated LM exposed at intercellular gap area. Immunohistochemistry revealed that LM abundantly existed in the cytosol of human portal and hepatic vein endothelial cells. These data suggest that LM can mediate from tethering to spreading of colon cancer cells under the blood flow and plays an essential role in haematogeneous metastasis. © 1999 Cancer Research Campaign


British Journal of Cancer (1999) 80(12), 1927–1934
© 1999 Cancer Research Campaign
Article no. bjoc.1999.0622

### Laminin mediates tethering and spreading of colon cancer cells in physiological shear flow

**J Kitayama[1], H Nagawa[1], N Tsuno[2], T Osada[2], K Hatano[1], E Sunami[1], H Saito[3] and T Muto[1]**

[1]Department of Surgery, Division of Surgical Oncology, [2]Department of Transfusion Medicine, [3]The Surgical Center, The University of Tokyo, Hongo 7-3-1, Bunkyo-ku, Tokyo 113, Japan

**Summary** Under the physiological shear condition, cultured colon cancer cells bound to laminin (LM), but not to fibronectin or vitronectin. Most of the tethered cells did not roll, but arrested immediately and spread within 10–30 min on LM under the continuous presence of shear flow. The tethering of Colo201 was partially inhibited by monoclonal antibodies (mAbs) to α6 integrin and a combination of mAbs to β1 and β4 integrins, but not by mAb to 67KD laminin receptor. Some Colo201 cells still tethered at 4°C. This suggests that α6β1 and α6β4 integrins participate in Colo201 tethering on LM, although other non-integrin molecules play roles. In contrast, the spread of Colo201 was effectively inhibited by the mAbs to integrin α2, α6 and β1 chains. The effect of anti-α2 plus anti-α6 mAbs was almost equal to anti-β1, suggesting that Colo201 cells mainly use α2β1 and α6β1 integrins for spreading on LM. When the cells were perfused on subconfluent endothelial cells (HUVEC) cultured on LM, they did not tether on HUVEC but did on coated LM exposed at intercellular gap area. Immunohistochemistry revealed that LM abundantly existed in the cytosol of human portal and hepatic vein endothelial cells. These data suggest that LM can mediate from tethering to spreading of colon cancer cells under the blood flow and plays an essential role in haematogeneous metastasis.

**Keywords**: metastasis; laminin; shear flow; α6 integrin

Haematogenous metastasis is an important factor affecting the prognosis of patients with colorectal cancer (Levin and Raijman, 1995). The attachment of circulating cancer cells to the vessel wall in target organs is one of the earliest events initiating tumour metastasis (Honn and Tang, 1992; Albelda, 1993; Gutman and Fidler, 1995). Previous studies have demonstrated that the attachment is mediated by various adhesion proteins, such as E-selectin and its counter receptors, sialyl Lewis-x (sLe-x) and sialyl Lewis-a (sLe-a), (Dejana et al, 1992; Takada et al, 1993; Irimura, 1994), VLA-4-VCAM-1 (Rice and Bevilacqua, 1989; Martin-Pandura et al, 1991), thrombospondin (Tuszynski et al, 1987; Walz, 1992), CD44 variants (St John et al, 1990; Gunthert et al, 1991) and other extracellular matrix (ECM) proteins (Humphries et al, 1986; Iwamoto et al, 1987; Liotta, 1991).

Leucocyte infiltration in inflammatory tissue or homing to lymphoid organs is a similar biological phenomenon as hematogenous metastasis of cancer cells, in terms of the interaction with local endothelium. The molecular mechanisms of the leucocyte emigration have been extensively studied, and it has been generally accepted that the initial tethering and rolling are supported by selectin-carbohydrate interactions, whereas integrins can not bind to their ligands under shear flow conditions but function mainly at the later steps (Butcher, 1991; Springer, 1994). In contrast to leucocytes, the effect of shear stress has not been carefully evaluated in the process of cancer cell attachment to vascular EC, although some adhesion mechanisms seem to be shared with cancer cells and leucocytes in static (Rice and Bevilacqua, 1989; Takada et al, 1993). Here, in this study, we used a flow adhesion assay system (Lawrence and Springer, 1991; Kitayama et al, 1997), and demonstrate that colon cancer cells tether, arrest and spread on immobilized laminin (LM) under the continuous presence of shear flow and that the each step is mediated by different LM receptors.

*Received 23 September 1998*
*Revised 12 January 1999*
*Accepted 26 January 1999*

*Correspondence to:* J Kitayama

### MATERIALS AND METHODS

### Reagents, antibodies and preparation of adhesion substrates

Fibronectin (FN), vitronectin (VN) and albumin (HSA) purified from human serum, as well as LM purified from human placenta were purchased from Calbiochem (La Jolla, CA, USA). These ECM proteins were diluted to 10–50 μg ml$^{-1}$ with phosphate-buffered saline (PBS), pH 7.4. Then, 20 μl of these solutions were spotted on polystyrene plates for 2 h at 37°C and blocked with 20 μg ml$^{-1}$ HSA for more than 48 h.

Monoclonal antibodies (mAbs) to integrin α1 (HP2B6, mIgG1) and α6 (GoH3, rat IgG2a) were purchased from Immunotech (Marseille, France). mAbs to α2 (P1H5, mIgG1) and α3 (P1B5, mIgG1) were from Telios Pharmaceuticals (La Jolla, CA, USA). Anti-β1 mAb (4B4, mIgG1) and control mouse-IgG1 or rat-IgG2a were obtained from Coulter (Hialeah, FL, USA) and anti-β4 mAb (UM-A9) was from Chemicon (Temecula, CA, USA). mAb to 67KD laminin receptor (MLuC5) was a generous gift from Dr Silvie Menard (Milan, Italy). mAb to LM (HL-4H3) recognizing P1 fragment of LM molecule was from Fuji Chemical Co. (Takaoka, Japan).

### Cell preparation

All the colon cancer cell lines, Colo201, Colo320, HT29, DLD-1 and WiDr were obtained from American Type Culture Collection (ATCC, Rockville, MD, USA) and maintained in Dulbecco's modified Eagle's medium (DMEM) with 10% fetal calf serum (FCS). HT29, DLD-1 and WiDr were detached from plastic flasks by the addition of 0.02% EDTA containing PBS + 0.25% trypsin, washed twice and incubated for at least 1 h at 4°C before the measurement of adhesion. All the cells were warmed up to 37°C just before the experiments. HUVEC were isolated from normal human umbilical cord and cultured as previously described (Kitayama et al, 1997).

### Laminar flow assay

A parallel-plate flow chamber (Kitayama et al, 1997) was mounted on the coated substrates, and placed on the stage of a phase contrast microscope and monitored with a 10× objective. Wall shear stress was calculated as previously described (Lawrence and Springer, 1991). The flow chamber and the optical area of the microscope were covered with a plastic box and the temperature of the inside was strictly maintained at 37°C. One million ($1 \times 10^6$) cells were suspended in 0.1 ml Hank's balanced salt solution (HBSS) with 10 mM HEPES (pH 7.4), kept at 4°C, and diluted with 1.0 ml of 37°C assay medium (HBSS with 10 mM HEPES including 2 mM calcium ion ($Ca^{2+}$), 1 mM magnesium ion ($Mg^{2+}$), and 10% FCS, pH 7.4) just before the experiments. Then, the cell suspensions were perfused on the substrates for 2 min through the flow chamber using an automated syringe pump (Harvard Apparatus, Natick, MA, USA) attached to the outlet side. For blocking experiments, the mAbs were added to $1 \times 10^6$ cell suspensions in 0.1 ml HBSS + HEPES, at a final concentration of 25 μg $ml^{-1}$ in each mAb. After 1 h incubation on ice, the cells were diluted with 1.0 ml assay medium and used for flow experiments.

Cells interacting with the substrate during flow were analysed from the images videotaped with a TEC-470 CCD video camera (Hamamatsu Photonics, Hamamatsu, Japan) and a Victor CVD-1000 recorder. In some experiments, after 2 min perfusion of cell suspension, the medium was perfused over the substrates for another 30 min, and the change in shape of the attached cells was monitored by videotape recorded in a time-lapse fashion.

### Evaluation of tethering, spreading and statistics

Tethered cells were defined as cells that maintained an adhesive interaction with the substrate for at least 1 second. The number of attached cells was counted over the 2 min after the flow became stable, and attached cells in three different fields were analysed. Cells that did not spread on the substrates were detected as bright cells with a round shape, whereas spread cells were observed as dark and enlarged figures under the phase contrast microscope. Thus, the two cell types were easily distinguished on the monitor screen. The *P*-values were calculated by paired Student's *t*-test and differences with $P < 0.05$ were considered to be significant.

### Immunohistochemistry

Frozen tissue sections of human normal liver were obtained from the donor for liver transplantation operation performed in the Hospital of the University of Tokyo. The sections were fixed with acetone and incubated with 5 μg $ml^{-1}$ of anti-LM mAb. Then, the reaction was carried out using ABC kit (Vector Laboratories, Burlingame, CA, USA) and peroxidase was developed with diaminobenzidine (DAB) and hydrogen peroxide ($H_2O_2$). The sections were counterstained with haematoxylin. For examination at higher magnifications, the sections were stained with the anti-LM mAb and fluorescein conjugated anti-mouse IgG, and observed with a confocal laser microscope (Fluoroview, Olympus, Tokyo, Japan).

### RESULTS

### Immobilized laminin supports tumour cell adhesion under shear flow

Extracellular matrix proteins, FN, LM and VN were coated on plastic plates at 10 μg $ml^{-1}$, and the adhesiveness of the colon cancer cells was examined under shear flow. At the shear of 1.0 dyn $cm^{-2}$, only a few cancer cells bound to FN and VN as well as control HSA. However, a considerable number of cells tethered and accumulated on LM coated at the same condition in all five cell lines (Figure 1A). The number of tethering Colo201 was largely decreased as the shear increased more than 1.5 dyn $cm^{-2}$, and no cells tethered at 3.0 dyn $cm^{-2}$ (Figure 1B). When the concentration of coating substrates was increased to 50 μg $ml^{-1}$, the number of Colo201 bound on LM increased almost as twice in each shear condition, whereas tetherings to FN and VN were negligible (Figure 1B). Interestingly, the majority of Colo201 tethered to LM did not roll but arrested immediately after the initial contact with LM, that was a marked contrast to leucocytes rolling on selectin substrates (Figure 2). Under the shear above 2.0 dyn $cm^{-2}$, some of the tethered Colo201 rolled for a short distance but detached to the flow stream in a few seconds, and thus no cells showed continuous rolling on LM. Other four cancer cells showed similar adhesion behavior as Colo201 (data not shown).

### Tethered colon cancer cells spread on immobilized laminin within minutes

After the attachment assay, the tethered and arrested cells on LM were subsequently subjected to continuous shear flow with warmed medium, and the shape change was observed for an additional 30 min. As shown in Figure 3, most of the tethered DLD-1 and HT29 cells were spread and flattened within the following 10–15 min under the continuous presence of 1.0 dyn $cm^{-2}$ shear. Colo201 usually grew at non-adhesive fashion under static culture condition. However, approximately 90% of Colo201 tethered on LM were flattened within the following 30 min under the same shear flow.

### Tethering and spreading on LM is partially mediated by α6β1 and α6β4 integrins

Many molecules, such as integrins and carbohydrates, are identified as receptors for LM (Mecham, 1991). Among the integrins, α1β1 (Hall et al, 1990), α2β1 (Elices and Hemler, 1989; Languino et al, 1989; Lotz et al, 1990), α3β1 (Gehlsen et al, 1992), α6β1 (Sonnenberg et al, 1988; Lotz et al, 1990) and α6β4 (Lotz et al, 1990; Lee et al, 1992) integrins have been reported to mediate binding to LM in static condition. To determine which integrins function in flow condition, we examined the adhesion behaviour

*© 1999 Cancer Research Campaign*

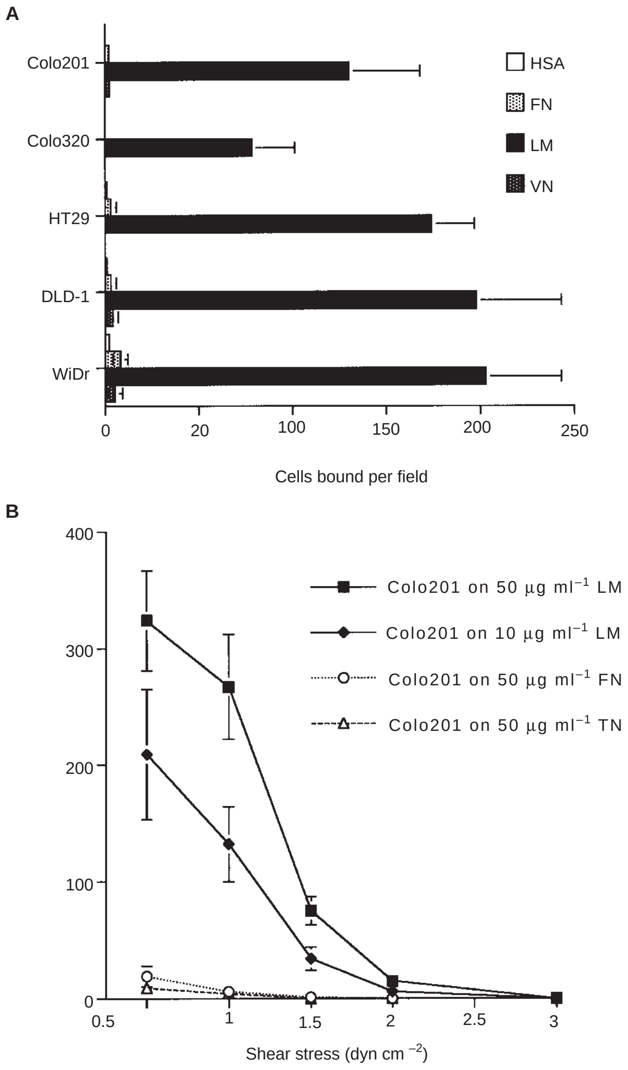

**Figure 1** Tethering activity of colon cancer cells on immobilized ECM proteins. Fibronectin (FN), vitronectin (VN), laminin (LM) and human serum albumin (HSA) were immobilized at the concentration of 10 µg ml$^{-1}$ (**A, B**) or 50 µg ml$^{-1}$ (**B**) as described in Materials and Methods. One million of Colo201, Colo320, HT29, DLD-1, WiDr suspended in 1.0 ml of assay medium were perfused on these substrates for 2 min at 1.0 dyn cm$^{-2}$ (**A**) or various shears (**B**), and the number of cells accumulated in three different areas was counted at the end of perfusion under a microscope in each experiment. Each value is mean ± s.d. of three different experiments

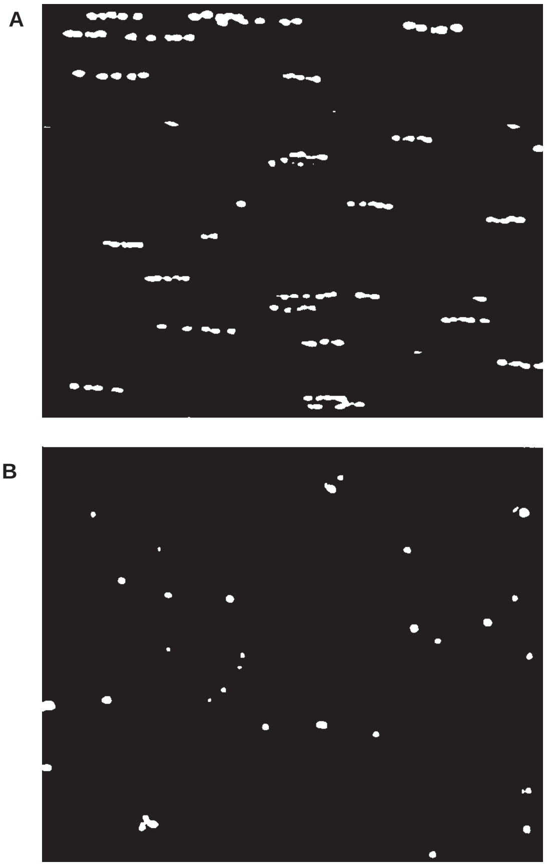

**Figure 2** Post-tethering behaviour of PMN on E-selectin (1 µg ml$^{-1}$) (**A**) and Colo201 on LM (10 µg ml$^{-1}$) (**B**) under the shear of 1.0 dyn cm$^{-2}$. Five consecutive images were captured every 2 seconds and superimposed with imaging analyser software (Adobe Premier, with a Macintosh computer). With flow from left to right, PMN rolled on E-selectin with a mean velocity of 13.7 µm per s, that was detected as five bright spots located linearly. Whereas none of the tethering Colo201 were displaced more than 1 cell diameter in the 10 seconds of Colo201 pretreated with functionally blocking mAbs to these integrins. As shown in Table 1, the number of tethering Colo201 was partially reduced to 60% ($P < 0.05$, $n = 4$) by the pretreatment with anti-α6 integrin mAb (GoH3). None of the mAbs to α1, α2 and α3 chains decreased the number of tethering Colo201. Moreover, none of the three mAbs induced further inhibitory effects of anti-α6 integrin mAb when used together with the mAb. Similarly, anti-β4 integrin mAb and anti-β1 integrin mAb alone showed no significant inhibition. However, when anti-β1 and anti-β4 mAbs were used together, the tethering of Colo201 was significantly inhibited to the same level as anti-α6 mAb (69% of control, $P < 0.01$, $n = 4$). This inhibition was specific since the addition of the same concentration of control mouse-IgG1 (50 µg ml$^{-1}$) have no significant effects. Neither anti-β1 nor anti-β4 augments the inhibitory effect of anti-α6 even when used together (data not shown). This suggests that the tethering of Colo201 to LM is partially mediated by both α6β1 and α6β4. However, neither anti-β1 nor anti-β4 mAbs alone produced any significant inhibition even used at the concentration of 100 µg ml$^{-1}$ (data not shown).

The effects of these mAbs on cell spreading were contrasted. Eighty-eight per cent of tethered Colo201 were flattened on LM after 30 min perfusion. Anti-α6 mAb, that partially reduced tethering cells, also significantly decreased the percentage of spread cells. The percentages of spread cells in total tethers of anti-α6 treated Colo201 was 61% (75% of control, $P < 0.05$, $n = 4$). Anti-α2 mAb, although showed no effect on tethering, strongly decreased the spreading of Colo201 to 38% (43% of control, $P < 0.05$, $n = 4$). Moreover, if anti-α2 mAb was used with anti-α6 mAb, the spreading rate was further decreased to 18% (20% of control, $P < 0.05$, $n = 4$). Anti-β1 integrin effectively inhibited the spreading of Colo201 to 15% (17% of control, $P < 0.01$, $n = 4$), that was almost the same level as when anti-α2 and anti-α6 mAbs were used together. In contrast, mAbs to α1 and α3 integrins showed no significant effect on spreading whether used alone or together with anti-α6 mAb. When Colo201 were pretreated with anti-β4 and anti-β1 mAbs, the percentages of spreading were at the similar level as Colo201 treated with anti-β1 alone (16 ± 6.7%

© 1999 Cancer Research Campaign

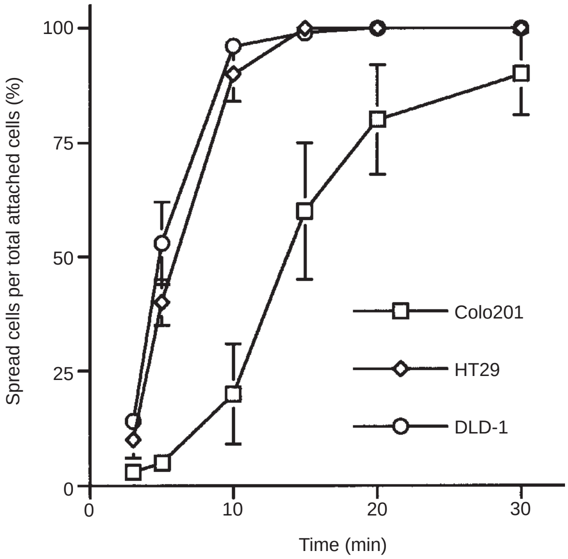

**Figure 3** Spreading of tethered cells on LM. Three colon cancer cells were perfused on immobilized LM (10 μg $ml^{-1}$) at 1.0 dyn $cm^{-2}$ for 2 min. Then, perfusion of the chamber was continued with warmed assay medium at the same speed for another 30 min, and the shape changes of the tethered cells were monitored on a screen. Total tethers were counted at the time points of 2 min after the initiation of perfusion. Then, all the tethered cells at the time points were marked on screen and whether the cells spread or remained as round shape within another 30 min was examined in each tether. The cells tethered after 2 min were neglected in this analysis. Data are mean ± s.e.m. of two separate experiments

**Table 1** Inhibition of tethering and spreading of Colo201 on LM in shear flow

| No treatment | Total tethers (cells/field)[a] | Percentage of spread cell (%)[b] |
|---|---|---|
| (–) | 127 ± 45 (100%) | 88 ± 10 (100%) |
| Anti-α1 | 136 ± 32 (> 100%) | 88 ± 16 (100%) |
| Anti-α2 | 130 ± 22 (> 100%) | 38 ± 16 (43%)[c] |
| Anti-α3 | 130 ± 13 (> 100%) | 86 ± 16 (98%) |
| Anti-α6 | 76 ± 20 (60%)[c] | 61 ± 16 (75%)[c] |
| Anti-β1 | 121 ± 41 (95%) | 15 ± 8.1 (17%)[d] |
| Anti-β4 | 118 ± 23 (93%) | 88 ± 9.1 (100%) |
| Anti-α1+anti-α6 | 84 ± 14 (63%)[c] | 60 ± 4.4 (68%)[c] |
| Anti-α2+anti-α6 | 80 ± 7.5 (66%)[c] | 18 ± 2.2 (20%)[c] |
| Anti-α3+anti-α6 | 90 ± 11 (71%)[c] | 51 ± 6.4 (60%)[c] |
| Anti-β1 +anti-β4 | 88 ± 37 (69%)[d] | 16 ± 6.7 (18%)[d] |
| Control mouse IgG1 | 132 ± 29 (> 100%) | 89 ± 2.5 (> 100%) |
| Control rat IgG2a | 144 ± 48 (> 100%) | 90 ± 5.8 (> 100%) |
| Control mouse IgG1[e] (50 μg ml)[1] | 142 ± 20 (> 100%) | 86 ± 3.8 (98%) |
| Control mouse IgG1 + rat IgG2a | 124 ± 48 (98%) | 92 ± 5.8 (> 100%) |
| 4°C | 59 ± 18 (46%)[d] | 0 (0%)[d] |
| 0.02% EDTA | 0 (0%)[d] | 0 (0%)[d] |

Colo201 cells treated with each mAbs at the concentration of 25 μg $ml^{-1}$ (or 50 μg $ml^{-1}$)[e] as described in Materials and Methods were perfused for 30 min in 1.0 dyn $cm^{-2}$. EDTA was used at a final concentration of 0.02%. In the experiments at 4°C, the flow chamber and LM-coated plate as well as the assay medium were cooled with ice before perfusion. [a]The number of tethered cells per field at 2 min after the initiation of perfusion. [b]The number of spread cells/total tethers at the end of perfusion. Each value is mean ± s.d. of four different experiments. Quoted numbers show the relative percentages to control without any treatment. [c]$P < 0.05$, [d] $P < 0.01$.

vs 15 ± 8.1%, $P > 0.05$, $n = 4$). The anti-β4 mAb did not augment the inhibitory effect of anti-α6 mAb on Colo201 spreading when used together (data not shown). Those data indicate that the step of cell spreading is mainly mediated by α2β1 and α6β1 integrins and suggest that α6β4 integrin does not have a major role for this step. Many Colo201 still tethered on LM even after the blocking of all these integrins. No cells tethered on LM when $Ca^{2+}$ was depleted from the assay medium by the addition of 0.02% EDTA. At 4°C, however, 46% of Colo201 examined at 37°C still tethered on LM (Table 1). This strongly suggests that another non-integrin receptor is involved in the tethering to LM. A 67 kDa monomeric polypeptide was reported to be a high affinity laminin receptor (Rao et al, 1983). Then, we used the functionally blocking mAb to the 67 kDa laminin receptor (MLuC5) (Van den Brule et al, 1996) for this flow experiment. However, the mAb did not show any inhibitory effects on Colo201 tethering whether used alone or with anti-α6 mAb (Table 2).

**Table 2** Effect of mAb to 67KD LR on Colo201 tethering on LM in shear flow

| No Treatment | Total tethers (cells/field) |
|---|---|
| (–) | 174 ± 22 (100%) |
| Anti-67KD LR | 180 ± 41 (>100%) |
| Anti-α6 | 15 ± 29 (65%)[a] |
| Anti-α6+anti-67KD LR | 131 ± 19 (75%)[a] |

Colo201 cells treated with each mAbs at the concentration of 25 μg $ml^{-1}$ were perfused and analysed as Table 1. Each value is mean ± s.d of three different experiments. [a]$P < 0.05$.

### Colon cancer cells did not tether on resting HUVEC but did tether on intercellular LM

Colo201 were perfused on HUVEC monolayer cultured on LM. As shown in Figure 4A, only a few cells tethered on resting HUVEC with confluent state. When the cells were perfused on sub-confluent HUVEC, few cells attached on HUVEC, while many cells tethered and accumulated at the intercellular gap area where the precoating of LM was supposed to be exposed to flowing cells (Figure 4B). This was confirmed by the fact that the accumulation was partially decreased by anti-α6 integrin mAb (Figure 4A).

### LM is abundantly expressed in endothelial cells of small portal and hepatic veins of human liver

LM mainly exists in the subendothelial basement membrane (Chung et al, 1979; Timpl et al, 1979), but it is not clear whether a considerable amount of LM exists in the area exposed to blood flow under physiological conditions. We tried the immunohistochemical staining of normal human liver tissue with anti-LM mAb recognizing the P1 epitope (Fujimoto, 1990). In addition to the connective tissue of Glisson, EC of the portal and central vein were strongly stained with anti-LM mAb (Figure 5 A,B). Sinusoidal EC were only partially stained by anti-LM mAb. This suggests the possibility that LM molecules are also expressed in the luminal side of small portal and hepatic veins.

 *© 1999 Cancer Research Campaign*

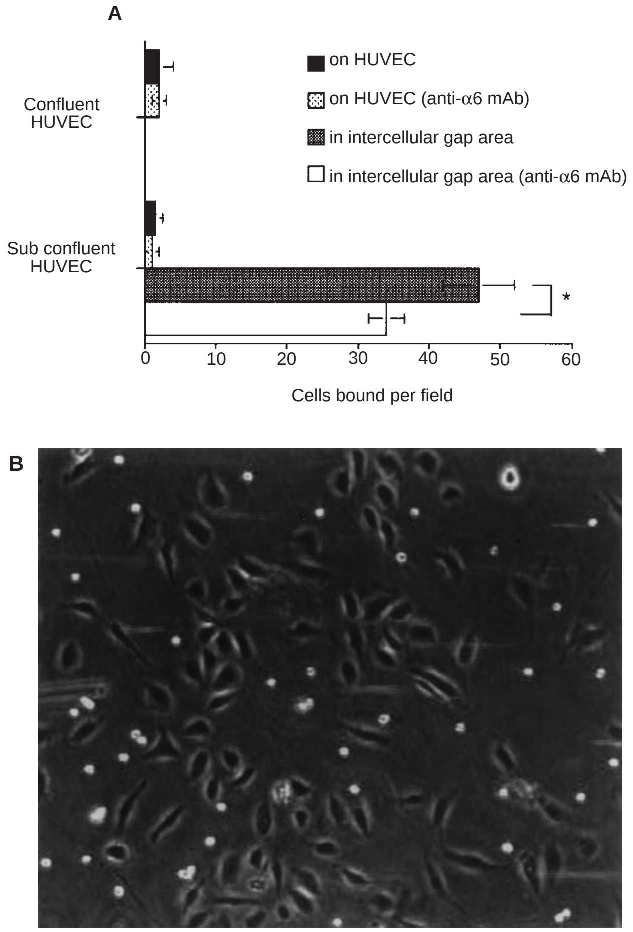

**Figure 4** Tethering of Colo201 to HUVEC or LM coated on plastic plates. Half million ($5 \times 10^5$) or $5 \times 10^4$ HUVEC were cultured on plastic plate (60 mm diameter) coated with LM (10 μg $ml^{-1}$). Two days later those HUVEC became confluent or sub-confluent state respectively. On these HUVEC monolayers, Colo201 cells were perfused as in Figure 1, and the tethered cells on HUVEC or at the bare area without HUVEC were separately counted in three different fields. mAb treatment was performed as Table 1. Data are mean ± s.d. in a representative experiment. *$P < 0.05$, $n = 3$. (**B**) Image at the end of perfusion on subconfluent HUVEC. Most of the Colo201 cells that tethered and arrested in flow were detected at the bare area between each HUVEC but not on the HUVEC

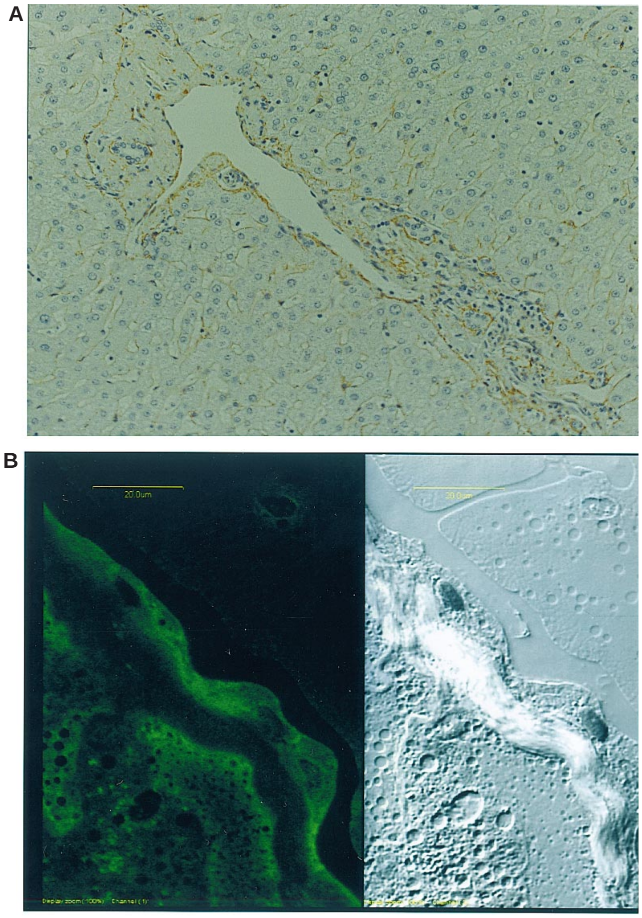

**Figure 5** Endothelial cells of small portal and hepatic veins in human liver abundantly expressed LM molecule. (**A**) Frozen sections of normal liver were stained with anti-LM mAb (HL-4H3) using ABC method and DAB development. (× 400). (**B**) The same sections were stained with FITC-labelled secondary mAb, and observed with a confocal laser microscope (Left: under fluorescein beam, Right: under normal light) (× 1400). Cytosol of small portal vein EC was brightly stained

### DISCUSSION

The lodgement of circulating cancer cells in the vascular bed of secondary organ is an essential step in metastatic process. This attachment must occur in the continuous presence of shear stress, similar to leucocyte tethering and rolling on inflammatory endothelium. In this study, we used five colon cancer cell lines as the models for liver metastasis of colon cancer. All the cell lines were established from human colon cancer tissues and had metastatic potential when injected intravenously. Here, we observed that all the five colon cancer cells tethered on immobilized LM substrate under physiological shear condition. Moreover, those tethered cells arrested immediately and spread within minutes order after the initial contact with LM. Tözeren et al (1994) has already reported that human epithelial and carcinoma cells tethered and rolled on the LM in the presence of laminar flow possibly through α6β4 integrin. In that study, they used relatively higher shear condition (> 3.5 dyn $cm^{-2}$) and described that the tethering cells rolled at the comparable velocity to that of leucocytes on P-selectin (Lawrence and Springer, 1991). In our study, however, all the tethering cancer cells did not roll but abruptly arrested at the shear below 1.5 dyn $cm^{-2}$. The ‘rapid sticking’ is specific for LM substrates, since these cancer cells showed rolling on E-selectin substrates (data not shown). Some tethers rolled for several seconds before the detachment to the flow at higher shear conditions, and no cells tethered under the shear above 3.0 dyn $cm^{-2}$ even the LM was coated at very high concentration. The exact reason of the difference between these two studies is not clear, but supposed to be attributed to many differences in experimental condition. One possible explanation is that LM purified from Englebreth–Holm–Swarm tumour used in the previous study has some differences in molecular structure from placenta-derived LM in ours. The difference in LM coating efficiency between on glass and on polystyrene may be related with the discrepancy. However, the adhesion pattern we observed in this study has already been described in mononuclear cells or eosinophils perfused on purified VCAM-1, MAdCAM-1, or activated HUVEC monolayer (Luscinskas et al, 1994; Alon et al, 1995;

© 1999 Cancer Research Campaign 

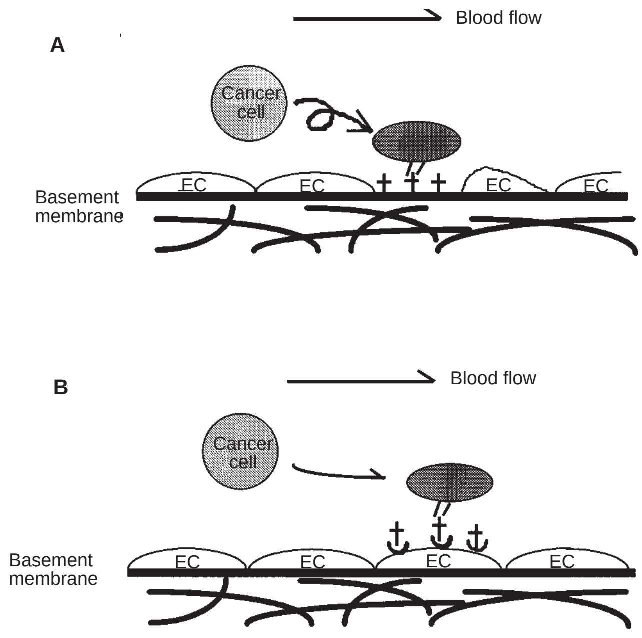

**Figure 6** Possible situations when LM functions as tethering molecule for cancer cells. (**A**) Subendothelial LM molecule is exposed to blood flow where EC are damaged and detached from the basement membrane. (**B**) LM was produced, bound to LM receptors, and presented on the luminal surface of certain EC. †: LM molecule, // and μ: LM receptors, EC: endothelial cell

Berlin et al, 1995; Kitayama et al, 1997). Those studies have shown that the rapid sticking is mediated by α4 integrins, and suggested that α4 integrin can immediately form tighter bonds than selectins that are strong enough to be resistant to the continuous shear force. Our data suggest that the cancer cells utilize certain adhesion receptors for LM that has similar kinetics in bond formation as α4 integrin. If the LM mediate the rolling interaction as described by Tozeren et al (1994) cancer cells require another factor to support cell arrest for metastasis formation. However, our finding suggests a possibility that a single cancer cell circulating in blood can develop metastasis only through the interaction with LM.

LM is one of the most abundant constituents of the basement membrane and regulates cell–ECM interactions such as adhesion, migration, growth and differentiation in various cell types (Chung et al, 1979; Timpl et al, 1979). The receptor for LM is not fully characterized yet. Integrins are the major receptors for ECM proteins. VLA-1 (α1 β1), VLA-2 (α2 β1), VLA-3 (α3 β1), VLA-6 (α6 β1), VLA-7 (α7 β1) and α6 β4 integrins have been reported to bind LM (Mecham, 1991). Our blocking experiments using Colo201 showed that tethering to LM was partially inhibited by anti-α6 mAb. Anti-β1 and anti-β4 mAbs alone showed no significant effect on tethering. The blocking results are totally consistent with the previous results by Tözeren et al (1994). In that work, however, they showed that cells expressing integrin β4 chain bound to LM in flow while cells lacking β4 integrin did not have any affinity to LM, and thus concluded that the tethering to LM was dependent on α6β4. In this study, however, we used anti-β1 and anti-β4 mAbs together, and found that they produced the same level of inhibition as anti-α6 mAb. This suggests that α6β1 as well as α6β4 have a contribution on Colo201 tethering on LM. The reason why either mAb alone did not show significant effect may be explained by that the contributions of each integrin on Colo201 tethering are too small to be recognized by the blockade of each integrin alone.

Total blockade in α6β1 and α6β4 integrins did not lead the complete inhibition in Colo201 tethering. The tethering to LM was totally blocked by $Ca^{2+}$ depletion, whereas some still tethered at 4°C condition (46% of control). From these data, it is unlikely that other LM binding integrins such as α7β1 (Kramer et al, 1991) play roles in Colo201 tethering, since all the integrin mediated adhesion should be abolished at 4°C. Then, we asked the contribution of a 67 kDa monomeric polypeptide reported as a high affinity laminin receptor (Rao et al, 1983). The expression of this receptor or its precursor on cancer cells has been reported to be correlated with malignant potential in various cancers, and induction of this 67 kDa receptor was reported to induce metastatic potential in non-metastatic counterparts (Castronovo et al, 1989; Coice et al, 1991; Van den Brule et al, 1996; Viacava et al, 1997). Although, the mAb to 67 kDa LM receptor partially inhibited the Colo201 adhesion to LM under static conditions (Viacava et al, 1997), it showed no significant inhibition in Colo201 tethering. Galectins have been reported to function as laminin receptors (Barondes et al, 1994; Van den Brule et al, 1995). However, the galectins are unlikely to mediate tethering under the flow condition, since the depletion of $Ca^{2+}$ with EDTA totally abolished the Colo201 tethering. Those data suggest that other uncharacterized laminin receptors participate in the colon cancer cell tethering in shear flow.

Another new finding in our study is that most of the tethering cancer cells were flattened within minutes' order when the shear flow was continuously subjected. Furthermore, the effects of anti-integrin mAbs on cell spreading were contrasted from those on tethering. The percentages of spread Colo201 in total tethers were significantly inhibited by anti-α6 and more strongly by anti-α2 mAbs, although the latter mAb showed no effect on Colo201 tethering. More than 80% of spreading was abolished by anti-β1 mAb. The effect of anti-α2 plus anti-α6 was additive and almost equal to anti-β1 integrin. In contrast, anti-β4 did not show clear effect to enhance the inhibitory effect of anti-β1 mAb even when used with anti-β1. No cells spread at 4°C. Those data clearly indicate that the post-tethering spreading on LM is mostly mediated by α2β1 and α6β1 integrins, while α6β4 did not have major role for cell spreading. In other words, colon cancer cells partially change the receptor usage from tethering to spreading on LM.

Recently, it has been shown that laminin have various isoforms, and each of them has specificity in its receptor usage, kinetics for adhesion function, and tissue distribution (Rousselle and Aumailley, 1994; Timpl and Brown, 1994; Tani et al, 1996). In particular, laminin 5 has been shown to mediate strong adhesion of various cell types including colon cancer cells mainly through α3β1 integrin (Rousselle and Aumailley, 1994; Orian-Rousseau et al, 1998). In our study, mAb to α3 showed no significant effect both on tethering and spreading of Colo201. However, the laminin we used in this study was purified from placenta that was consisted mainly of laminin 1, and thus the precise role of α3β1 integrin for metastasis formation needs to be investigated using purified form of laminin 5.

From our results, we can speculate two possible situations that the LM molecule function as tethering molecule for cancer metastasis (Figure 6). Although LM mainly exists at basement membrane, the subendothelial LM molecule can be exposed to blood flow when EC are damaged and detached from the basement membrane. In this situation, the LM molecule is supposed to trap the circulating cancer cells even in blood flow that results in the

 © 1999 Cancer Research Campaign metastasis formation in those areas (Figure 6A). Animal studies have shown that endothelial injury by drug (Orr et al, 1986), hyperoxia (Adamson et al, 1987), radiation (von Essen, 1991), or activated leucocytes (Orr and Warner, 1987) significantly promotes local arrest and metastasis in the lung. We found that Colo201 cells, if perfused on sub-confluent HUVEC, preferentially tethered at the bare area as shown in Figure 4B. From this finding, the preferential deposition of tumour cells in EC denuded areas in those animal studies is considered to be mediated through LM molecule.

Another possibility is that LM may be expressed on the luminal surface of EC in certain organs. Some EC have been shown to produce LM (Gospodarowicz et al, 1981; Tokida et al, 1990). Our staining experiment clearly showed that portal and hepatic veins in the normal liver strongly expressed LM molecules in their cytosol. In these vessels, therefore, LM is supposed to be presented on the luminal surface, since microvascular EC were reported to express LM receptors such as 67 kDa receptor or β1 integrins on their cell surface membrane (Yannariello-Brown et al, 1988; Cheng and Kramer, 1989). In fact, an electron microscopic study has demonstrated that LM is present on the luminal surface of capillaries in murine lung (Hilario et al, 1996). Those LM molecules can be recognized by another LM receptor on cancer cells, and thus may function as a tethering molecule in organ-specific metastasis (Figure 6B).

In summary, colon cancer cells showed tethering, arrest and subsequent spreading on immobilized LM under physiological shear flow. The tethering was partially mediated by α6β1 and α6β4, while the spreading was mostly by α2β1 and α6β1 integrins. Although LM has been proposed to have a strong association with the metastatic potential of various cancers (Hunt, 1989; Liotta, 1991; Honn and Tang, 1992), our study further emphasizes the role of this molecule in tumour metastasis. This interaction seems to be especially important when the metastasis develops in EC-damaged areas or in organs with LM-rich vessel system.

### ACKNOWLEDGEMENTS

We thank Dr Timothy A Springer for the critical reading of this manuscript. This work was supported partly by a Grant-in-Aid for Scientific Research from the Ministry of Education, Science, Sports and Culture of Japan and partly by a Grant from the Ministry of Health and Welfare of Japan.

### REFERENCES

Adamson IYR, Young L and Orr FW (1987) Tumor metastasis after hyperoxic injury and repair of the pulmonary endothelium. *Lab Invest* **57**: 71–77

Albelda SM (1993) Role of integrins and other cell adhesion molecules in tumor progression and metastasis. *Lab Invest* **68**: 4–17

Alon R, Kassner PD, Carr MW, Finger EB, Hemler ME and Springer TA (1995) The integrin VLA-4 supports tethering and rolling in flow on VCAM-1. *J. Cell Biol* **128**: 1243–1253

Barondes SH, Cooper DNW, Gitt MA and Leffler H (1994) Galectins: structure and function of a large family of animal lectins. *J Biol Chem* **269**: 20807–20810

Berlin C, Bargatze RF, von Andrian UH, Szabo MC, Hasslen SR, Nelson RD, Berg EL, Erlandsen SL and Butcher EC (1995) a4 integrins mediate lymphocyte attachment and rolling under physiologic flow. *Cell* **80**: 413–422

Butcher EC (1991) Leukocyte-endothelial cell recognition: Three (or more) steps to specificity and diversity. *Cell* **67**: 1033–1036

Castronovo V, Taraboletti G, Liotta LA and Sobel ME (1989) Modulation of laminin receptor expression by estrogen and progestins in human breast cancer cell lines. *J Natl Cancer Inst* **81**: 782–788

Cheng YF and Kramer RH (1989) Human microvascular endothelial cells express integrin-related complexes that mediate adhesion to the extracellular matrix. *J Cell Physiol* **139**: 275–286

Chung AE, Jaffe R, Freeman IL, Vergnes JP, Braginski JE and Carlin B (1979) Properties of basement-membrane-related glycoprotein synthesized in culture by a mouse embryonal carcinoma derived cell line. *Cell* **16**: 277–281

Coice V, Castronova V, Shmookler BM, Garbisa S, Grigioni WF, Liotta LA and Sobel ME (1991) Increased expression of the laminin receptor in human colon cancer. *J Natl Cancer Inst* **83**: 29–36

Dejana E, Martin-Padura I, Lauri D, Bernasconi S, Bani MR, Garofalo A, Giavazzi R, Magnani J, Mantovani A and Menard S (1992) Endothelial leukocyte adhesion molecule-1 dependent adhesion of colon carcinoma cells is inhibited by an antibody to Lewis fucosylate type I carbohydrate chain. *Lab Invest* **66**: 324–330

Elices MJ and Hemler ME (1989) The human integrin VLA-2 is a collagen receptor on some cells and a collagen/laminin receptor on others. *Proc Natl Acad Sci USA* **86**: 9906–9910

Fujimoto N (1990) One step sandwich enzyme immunoassay for human laminin using monoclonal antibodies. *Clin Chim Acta* **191**: 211–220

Gehlsen KR, Sriramarao P, Furcht LT and Skubitz APN (1992) A synthetic peptide derived from the carboxy terminus of the laminin A chain represents a binding site for a3b1 integrin. *J Cell Biol* **117**: 449–459

Gospodarowicz D, Greenberg G, Foidart JM and Savion N (1981) The production and localization of laminin in cultured vascular and corneal endothelial cells. *J Cell Physiol* **107**: 171–183

Gunthert U, Hofmann M, Rudy W, Reber S, Zoller M, Haubmann I, Matzku S, Wenzel A, Ponta H and Herrlich P (1991) A new variant of glycoprotein CD44 confers metastatic potential to rat carcinoma cells. *Cell* **65**: 13–24

Gutman M and Fidler IJ (1995) Biology of human colon cancer metastasis. *World J Surg* **19**: 226–234

Hall DE, Reichardt LF, Crowley E, Holley B, Moezzi H, Sonnenberg A and Damsky CH (1990) The $alpha_1/b_1$ and $alpha_6/b_1$ integrin heterodimers mediate cell attachment to distinct sites on laminin. *J Cell Biol* **110**: 2175–2184

Hilario E, Unda F, Perez-Yarza G, Alvarez A, Garcia-Sanz M and Alino SF (1996) Presence of laminin and 67 kDa laminin-receptor on endothelial surface of lung capillaries. An immunohistochemical study. *Histol Histopathol* **11**: 915–918

Honn KV and Tang DJ (1992) Adhesion molecules and tumor cell interaction with endothelium and subendothelial matrix. *Cancer Metastasis Rev* **11**: 353–375

Humphries MJ, Olden K and Yamada KM (1986) A synthetic peptide from fibronectin inhibits experimental metastasis of murine melanoma cells. *Science* **233**: 467–470

Hunt G (1989) The role of laminin in cancer invasion and metastasis. *Exp Cell Biol* **57**: 165–176

Irimura T (1994) Cancer metastasis determined by carbohydrate-mediated cell adhesion. *Adv Exp Med Biol* **353**: 27–34

Iwamoto Y, Robey FA, Graf J, Sasaki M, Kleinman HK, Yamada Y and Martin GR (1987) YIGSR, a synthetic laminin pentapeptide, inhibits experimental metastasis formation. *Science* **238**: 1132–1134

Kitayama J, Fuhlbrigge RC, Puri KD and Springer TA (1997) P-selectin, L-selectin, and a4 integrin have distinct roles in eosinophil tethering and arrest on vascular endothelial cells under physiological flow conditions. *J Immunol* **159**: 3929–3939

Kramer RH, Vu MP, Cheng Y-F, Ramos DM, Timpl R and Waleh N (1991) Laminin-binding integrin a7b1: functional characterization and expression in normal and malignant melanocytes. *Cell Reg* **2**: 805–817

Languino LR, Gehlsen KR, Wayner EA, Carter WG, Engvall E and Ruoslahti E (1989) Endothelial cells use alpha2 betal integrin as a laminin receptor. *J Cell Biol* **109**: 2455–2462

Lawrence MB and Springer TA (1991) Leukocytes roll on a selectin at physiologic flow rates: distinction from and prerequisite for adhesion through integrins. *Cell* **65**: 859–873

Lee EC, Lotz MM, Steele GD Jr and Mercurio AM (1992) The integrin $a^6b4$ is a laminin receptor. *J Cell Biol* **117**: 671–678

Levin B and Raijman I (1995) Gastroenterology (Philadelphia: Saunders)

Liotta L (1991) Tumor invasion and metastases – role of the extracellular matrix. *Cancer Res* **46**: 1–7

Lotz MM, Korzelius CA and Mercurio AM (1990) Human colon carcinoma cells use multiple receptors to adhere to laminin: Involvement of alpha 6 beta 4 and alpha 2 beta 1 integrins. *Cell Reg* **1**: 249–257

Luscinskas FW, Kansas GS, Ding H, Pizcueta P, Schleiffenbaum BE, Tedder TF and Gimbrone MA Jr (1994) Monocyte rolling, arrest and spreading on IL-4-activated vascular endothelium under flow is mediated via sequential action of L-selectin, b1-integrins, and b2-integrins. *J Cell Biol* **125**: 1417–1427

*© 1999 Cancer Research Campaign*

Martin-Pandura I, Mortarini R, Lauri D, Bernasconi S, Sanchez-Madrid F, Parmiani G, Mantovani A, Anichini A and Dejana E (1991) Heterogeneity in human melanoma cell adhesion to cytokine activated endothelial cells correlates with VLA-4 expression. *Cancer Res* **51**: 2239–2241

Mecham RP (1991) Laminin receptors. *Ann Rev Cell Biol* **7**: 71–91

Orian-Rousseau V, Aberdam D, Rousselle P, Messent A, Gavrilovic J, Meneguzzi G, Kedinger M and Simon-Assmann P (1998) Human colonic cancer cells synthesize and adhere to laminin-5. Their adhesion to laminin-5 involves multiple receptors among which is integrin alpha2betal. *Journal of Cell Science* **111**: 1993–2004

Orr FW and Warner DJA (1987) Effect of neutrophil-mediated pulmonary endothelial injury on the localization and metastasis of circulating Walker carcinima cells. *Invasion Metastasis* **7**: 183–196

Orr FW, Adamson IYR and Young L (1986) Promotion of pulmonary metastasis in mice by bleomycin-induced endothelial injury. *Cancer Res* **46**: 891–897

Rao CN, Barsky SH, Terranova VP and Liotta LA (1983) Isolation of a tumor laminin receptor. *Biochem Biophys Res Commun* **111**: 804–808

Rice GE and Bevilacqua MP (1989) An inducible endothelial cell surface glycoprotein mediates melanoma adhesion. *Science* **246**: 1303–1306

Rousselle P and Aumailley M (1994) Kalinin is more efficient than laminin in promoting adhesion of primary keratinocytes and some other epithelial cells and has a different requirement for integrin receptors. *J Cell Biol* **125**: 205–214

Sonnenberg A, Modderman PW and Hogervorst F (1988) Laminin receptor on platelets is the integrin VLA-6. *Nature* **336**: 487–489

Springer TA (1994) Traffic signals for lymphocyte recirculation and leukocyte emigration: the multi-step paradigm. *Cell* **76**: 301–314

St John T, Meyer J, Idzerda R and Gallatin WM (1990) Expression of CD44 confers a new adhesive phenotype on transfected cells. *Cell* **60**: 45–52

Takada A, Ohmori K, Yoneda T, Tsuyuoka K, Hasegawa A, Kiso M and Kannagi R (1993) Contribution of carbohydrate antigens sialyl Lewis A and sialyl Lewis X to adhesion of human cancer cells to vascular endothelium. *Cancer Res* **53**: 354–361

Tani T, Karttunen T, Kiviluoto T, Kivilaakso E, Burgeson RE, Sipponen P and Virtanen I (1996) a6b4 integrin and newly deposited laminin-1 and laminin-5 form the adhesion mechanisms of gactric carcinoma. *Am J Pathol* **149**: 781–793

Timpl R and Brown JC (1994) The laminins. *Matrix Biol* **14**: 275–281

Timpl R, Rohde H, Robey PG, Renard SI, Foidart JM and Martin GR (1979) Laminin; a glycoprotein from basement membranes. *J Biol Chem* **254**: 9933–9937

Tokida Y, Aratani Y, Morita A and Kitagawa Y (1990) Production of two variant laminin forms by endothelial cells and shift of their relative levels by angiostatic steroids. *J Biol Chem* **265**: 18123–18129

Tözeren A, Kleinman HK, Wu S, Mercurio AM and Byers SW (1994) Integrin alpha 6 beta 4 mediates dynamic interactions with laminin. *J Cell Sci* **107**: 3153–3163

Tuszynski GP, Rothman V, Murphy A, Siegler K, Smith L, Smith S, Karczewski J and Knudsen KA (1987) Thrombospondin promotes cell-substrate adhesion. *Science* **236**: 1570–1573

Van den Brule FA, Buicu C, Balder M, Sobel ME, Cooper DNW, Marschal, P and Castronovo V (1995) Galectin-1 modulates human melanoma cell adhesion to laminin. *Biochem Biophys Res Commun* **209**: 760–767

Van den Brule FA, Castronovo V, Menard S, Giavazzi R, Marzola M, Belotti D and Taraboletti G (1996) Expression of the 67kD laminin receptor in human ovarian carcinomas as defined by a monoclonal antibody, MLuC5. *Eur J Cancer* **32A**: 1598–1602

Viacava P, Naccarato AG, Collecchi P, Menard S, Castronovo V and Generoso B (1997) The spectrum of 67kD laminin receptor expression in breast carcinoma progression. *J Pathol* **182**: 36–44

von Essen CF (1991) Radiation enhancement of metastasis. *Clin Exp Metastasis* **9**: 77–104

Walz DA (1992) Thrombospondin as a mediator of cancer cell adhesion and in metastasis. *Cancer Metastasis Rev* **11**: 313–324

Yannariello-Brown J, Wewer U, Liotta L and Madri JA (1988) Distribution of a 69-kDa laminin binding protein in aortic and microvascular endothelial cells: modulation during cell attachment, spreading, and migration. *J Cell Biol* **106**: 1773–1786

 *© 1999 Cancer Research Campaign*
